# *UvHOG1* is important for hyphal growth and stress responses in the rice false smut fungus *Ustilaginoidea virens*

**DOI:** 10.1038/srep24824

**Published:** 2016-04-20

**Authors:** Dawei Zheng, Yi Wang, Yu Han, Jin-Rong Xu, Chenfang Wang

**Affiliations:** 1 State Key Laboratory of Crop Stress Biology for Arid Areas, College of Plant Protection, Northwest A&F University, Yangling, Shaanxi 712100, China; 2 Dept. of Botany and Plant Pathology, Purdue University, West Lafayette, IN 47907 USA

## Abstract

Rice false smut caused by *Ustilaginoidea virens* is one of the most important diseases of rice worldwide. Although its genome has been sequenced, to date there is no report on targeted gene deletion in *U. virens* and no molecular studies on genetic mechanisms regulating the infection processes of this destructive pathogen. In this study, we attempted to generate knockout mutants of the ortholog of yeast *HOG1* MAP kinase gene in *U. virens*. One *Uvhog1* deletion mutant was identified after screening over 600 hygromycin-resistant transformants generated by *Agrobacterium tumefaciens* mediated transformation. The *Uvhog1* mutant was reduced in growth rate and conidiation but had increased sensitivities to SDS, Congo red, and hyperosmotic stress. Deletion of *UvHOG1* resulted in reduced expression of the stress response-related genes *UvATF1* and *UvSKN7*. In the *Uvhog1* mutant, NaCl treatment failed to stimulate the accumulation of sorbitol and glycerol. In addition, the *Uvhog1* mutant had reduced toxicity on shoot growth in rice seed germination assays. Overall, as the first report of targeted gene deletion mutant in *U. virens*, our results showed that *UvHOG1* likely has conserved roles in regulating stress responses, hyphal growth, and possibly secondary metabolism.

*Ustilaginoidea virens* (Cooke) Takah (Teleomorph: *Villosiclava virens*) is the causal agent of rice false smut that threatens rice production worldwide^1^. It is an ascomycete that is closely related to *Claviceps purpurea* and its genome has been recently sequenced^2^. In infected rice kernels, seed development is inhibited and replaced with the formation of so-called smut balls that contain darkly-pigmented chlamydospores. In addition to causing yield losses, *U. virens* produces ustiloxins that have inhibitory effects on growth of plant seedlings and are harmful to the nervous system of animals^3^,^4^,^5^.

Although the success rate is relatively low, false smut symptom development can be observed by inoculation with conidia at the rice booting stage^6^. It has been shown that germ tubes of *U. virens* can enter and grow intercellularly in the filaments of rice heads. When the fungus reaches the base of filaments, hyphal growth extends upward from basal filaments to anther apex and finally encloses the floral organs to form the smut balls^5^,^7^. Due to *U. virens* infection, rice seed development is completely stopped^8^. However, molecular mechanisms regulating the plant infection processes of the rice false smut fungus are not clear. In fact, although its genome has been sequenced^2^, targeted gene deletion by homologous recombination has not been reported in *U. virens*. Nevertheless, *U. virens* is amenable to transformation and disruption mutants can be identified by random insertional mutagenesis^9^. In comparison with many other plant pathogenic fungi such as *Magnaporthe oryzae, Fusarium graminearum,* and *Ustilago maydis*^10^,^11^,^12^, the frequency of homologous recombination and gene replacement is low in *C. purpurea*^13^,^14^. It is possible that *U. virens* also has a relatively low homologous recombination frequency, making targeted gene knockout, an important approach to study gene functions in pathogenic fungi, less efficient in this important rice pathogen.

In the budding yeast *Saccharomyces cerevisiae*, the high osmolarity glycerol (HOG) response pathway consisting of the Ssk2/Ssk22-Pbs2-Hog1 MAP kinase (MAPK) cascade is important for survival under hyperosmotic conditions^15^,^16^. In filamentous fungi, the key components of this MAP kinase pathway are well conserved but their biological functions are not limited to responses to high osmolarity^17^,^18^. In general, Hog1 orthologs have been implicated in regulating plant infection, fungicide resistance, and responses to various environmental stresses, such as oxidative and cell wall stresses. However, its exact functions vary significantly among different fungal pathogens. For example, the Hog1 ortholog is important for plant infection in *Botrytis cinerea, Mycosphaerella graminicola,* and *F. graminearum,* but deletion of *OSM1* in *M. oryzae* or its ortholog in *Colletotrichum lagenarium* has no effect on penetration and virulence^19^,^20^,^21^,^22^,^23^. Whereas the *Bcsak1* and *sakA* mutants are hypersensitive to oxidative stress in *B. cinerea* and *A. fumigatus*^20^,^21^,^24^, the *Fghog1* and *Cshog1* deletion mutants are only slightly reduced in growth rate by H_2_O_2_ in *F. graminearum* and *Bipolaris sorokiniana*^20^,^25^. In *C. lagenarium*, *Neurospora crassa*, and other fungi, the *HOG* pathway plays a role in resistance to dicarboximide and phenylpyrrole fungicides. In *F. graminearum*, the FgHog1 MAP kinase pathway is also important for hyphal growth, the production of deoxynivanol (DON), a trichothecene mycotoxin, and sexual reproduction^20^.

Like many other filamentous fungi^18^, *U. virens* has the Hog1 and other two well-conserved MAP kinase cascades. However, none of them have been functionally characterized. In this study, we generated the *Uvhog1* mutant and characterized its defects. As the first report on generation of a targeted deletion mutant in *U. virens*, we found that this fungal pathogen has a relatively low homologous recombination frequency (<0.2%). The *Uvhog1* mutant was reduced in vegetative growth and conidiation but had increased sensitivities to hyperosmotic and cell wall stresses. Our results showed that *UvHOG1* likely has conserved roles in stress responses in *U. virens* and improvements are necessary to make molecular studies more efficient and routine.

## Results

### *U. virens* has a relatively low homologous recombination frequency

The predicted gene KDB17291.1 of *U. virens* is orthologous to the yeast *HOG1* and named *UvHOG1* in this study. To generate the *Uvhog1* deletion mutant, we first transformed the *UvHOG1* gene replacement construct generated by the double-joint PCR approach^26^ into protoplasts of the wild-type strain UV-8b^2^. After screening over 300 hygromycin-resistant transformants derived from at least five independent transformations, we failed to identify any *Uvhog1* deletion mutant, indicating that the homologous recombination efficiency is relatively low in *U. virens*, which is consistent with what has been observed in *C. purpurea*^14^.

**Figure 1:**
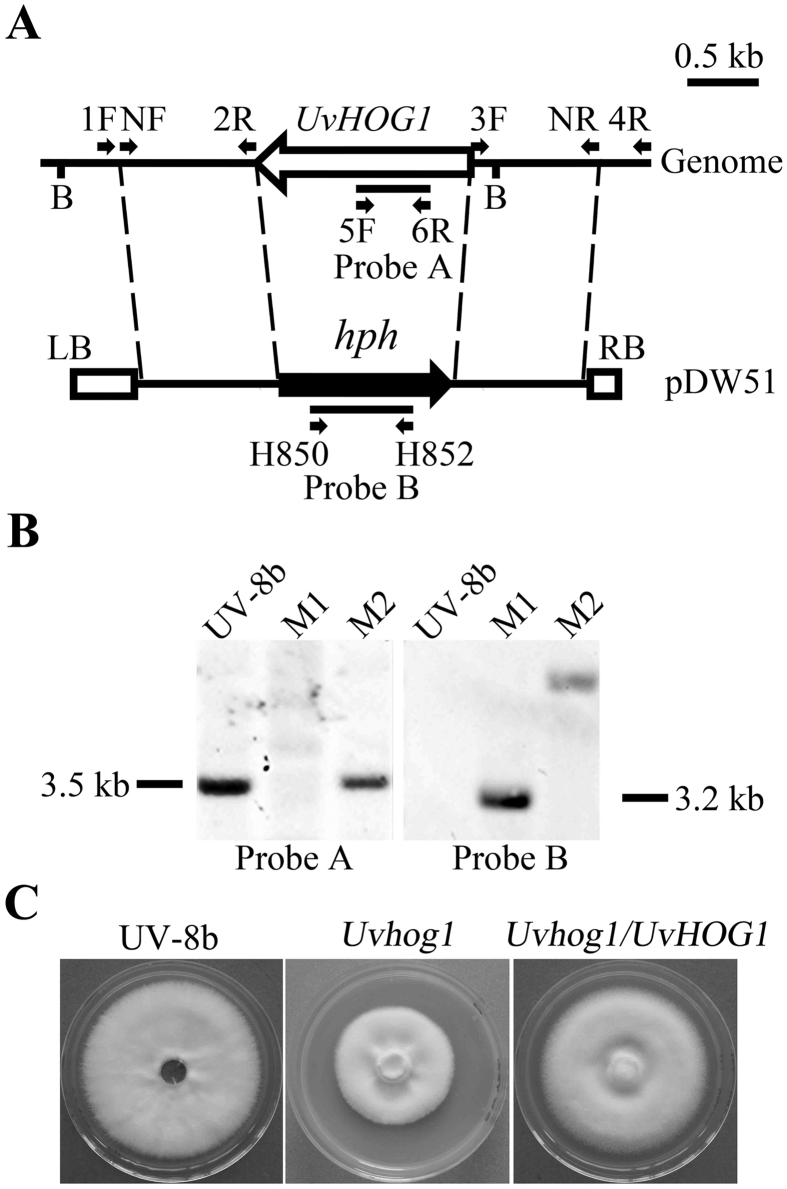
Generation of the *Uvhog1* mutant. (**A**) The *UvHOG1* locus and gene replacement construct. The *UvHOG1* and *hph* genes are marked with empty and black arrows, respectively. 1F, NF, 2R, 3F, 4R, 5F, 6R, and NR are primers used to amplify the flanking sequences or for mutant screening. B: *Bam*HI. LB: Left border; RB: Right border. (**B**) Southern blots of genomic DNA isolated from the wild type strain UV-8b, *Uvhog1* mutant M1, and an ectopic transformant M2 were hybridized with probe A (left) amplified with primers 5F and 6R or probe B (right) amplified with primers H852 and H850. All DNA samples were digested with *Bam*HI. (**C**) Colony morphology of the wild type, *Uvhog1* mutant, and complementary strain grown on 5xYEG plates.

Because *Agrobacterium tumafaciens*-mediated transformation (ATMT) is known to increase the frequency of homologous recombination^27^, we then cloned the *UvHOG1* gene replacement construct generated by double-joint PCR into the binary vector pCAMBIA1300^28^. The resulting vector was introduced into *A. tumafaciens* and used to transform conidia of UV-8b. A total of 619 hygromycin-resistant ATMT transformants were screened by PCR with primers (see Supplementary Table S1 online) located in the deleted region. One putative knockout mutant M1 was identified (Fig. 1A) and further confirmed by Southern blot hybridization (Fig. 1B). In the wild type and ectopic transformant M2, a 3.5-kb *Bam*HI band was detected with an *UvHOG1* fragment as the probe (Fig. 1B). The same probe had no hybridization signals in transformant M1. When hybridized with a fragment of the *hph* gene, a 3.2-kb band of the expected size derived from the gene replacement event was detected in the *Uvhog1* deletion mutant M1 (Fig. 1B). The wild type had no hybridization signals but ectopic transformant M2 had a 5.0-kb band (Fig. 1B). Therefore, the homologous recombination efficiency was as low as 0.16% for the *UvHOG1* gene in *U. virens*.

### *UvHOG1* is important for vegetative growth and conidiation

Because the *Uvhog1* mutant formed smaller colonies than the wild type (Fig. 1C), we assayed its growth rate on PDA, YT, and 5xYEG. On all the media tested, the *Uvhog1* mutant was slightly reduced in growth rate (Table 1), indicating that its defects in vegetative growth was independent of nutrient conditions.

We also assayed the production of conidia in liquid YT cultures^29^. Although it produced normal conidia, the *Uvhog1* mutant was significantly reduced in conidiation in comparison with the wild type (Table 1). Whereas the wild-type strain produced 8.2 × 10^5^ conidia/ml in 7-day-old cultures, the mutant produced less than 1.8 × 10^5^ conidia/ml under the same conditions.

For complementation assays, the entire *UvHOG1* gene (the coding region and its 1.5-kb promoter and 0.5-kb terminator sequences) was amplified from strain UV-8b and transformed into the *Uvhog1* mutant. The resulting complemented transformant C1 was rescued in the defects of *Uvhog1* mutant in hyphal growth and conidiation (Table 1). These results indicate that deletion of *UvHOG1* is directly responsible for the mutant phenotypes and the *UvHOG1* gene plays a role in hyphal growth and conidiation in *U. virens*.

### Deletion of *UvHOG1* results in defects in response to hyperosmotic stress

**Figure 2:**
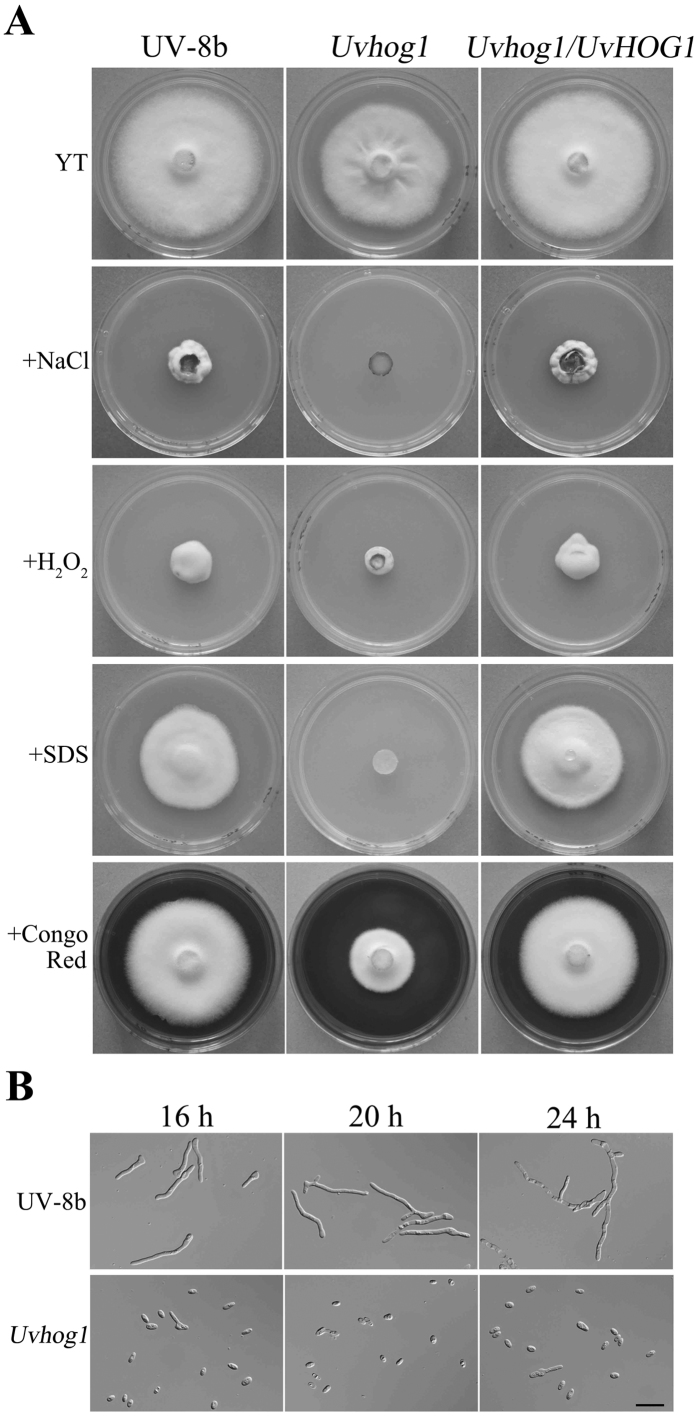
Growth and conidium germination of the *Uvhog1* mutant in the presence of different stresses. (**A**) The wild-type strain UV-8b, *Uvhog1* mutant, and complementary transformant were cultured on YT medium with or without 0.5 M NaCl, 0.07% H_2_O_2_, 0.03% SDS (w/v), and 70 μg/ml Congo red. Photographs were taken after incubation at 25 °C for 14 days. (**B**) Conidia of UV-8b and the *Uvhog1* mutant were incubated in YTS with 0.3 M NaCl for 16, 20, or 24 h. Bar = 20 μm.

Because the HOG pathway is well conserved in fungi for responses to hyperosmotic stress^17^,^18^, we assayed the defects of the *Uvhog1* mutant in growth on YT medium with 0.5 M NaCl or 1 M sorbitol. In the presence of 0.5 M NaCl, the *Uvhog1* mutant had no obvious growth after incubation for 14 days (Fig. 2A). Under the same conditions, the wild type was reduced in growth rate but still formed compact colonies (Fig. 2A; Table 2). We also assayed the effect of hyperosmotic stress on conidium germination. In the presence of 0.3 M NaCl, most of the wild-type conidia (72.1 ± 1.9%) germinated after incubation in YTS medium for 16 h but only 3.2 ± 1.3% mutant conidia germinated. Even after incubation for 24 h, only 5.0 ± 0.8% conidia produced germ tubes. Moreover, germ tube growth was stunted by NaCl treatment in the *Uvhog1* mutant (Fig. 2B; see Supplementary Table S2 online). Similar results were obtained with growth assays on medium with 1 M sorbitol. Treatments with 0.5 M NaCl or 1 M sorbitol resulted in over 99% reduction in colonial growth in the *Uvhog1* mutant (Table 2).

### *UvHOG1* is also important for responses to cell wall and membrane stresses but not oxidative stress

We also assayed the defects of the *Uvhog1* mutant in response to oxidative, cell wall and membrane stresses on YT medium. In the presence of 0.07% H_2_O_2_, both the wild type and *Uvhog1* mutant had a similar level of reduction in growth rate after incubation for 14 days (Fig. 2A; Table 2). On medium with 70 μg/ml Congo red, the growth rate was reduced by approximately 17% in the wild type but 50% in the *Uvhog1* mutant (Fig. 2A; Table 2). However, in the presence of 0.03% SDS (w/v), the *Uvhog1* mutant had no visible growth although the wild-type strain UV-8b still produced compact colonies (Fig. 2A). After incubation in YTS medium with 0.03% SDS for 12 h, most of the wild type conidia (76.2 ± 1.2%) germinated but less than 2.5 ± 1.0% *Uvhog1* mutant conidia produced germ tubes (see Supplementary Fig. S1 and Table S2 online). These results suggest that the *Uvhog1* mutant also had increased sensitivity to Congo red and SDS. Therefore, the *UvHOG1* pathway is involved in regulating responses to membrane and cell wall stresses, but not oxidative stress in *U. virens*.

### Deletion of *UvHOG1* affects the expression of *UvATF1*, *UvSKN7*, and *UvAP1*

**Figure 3:**
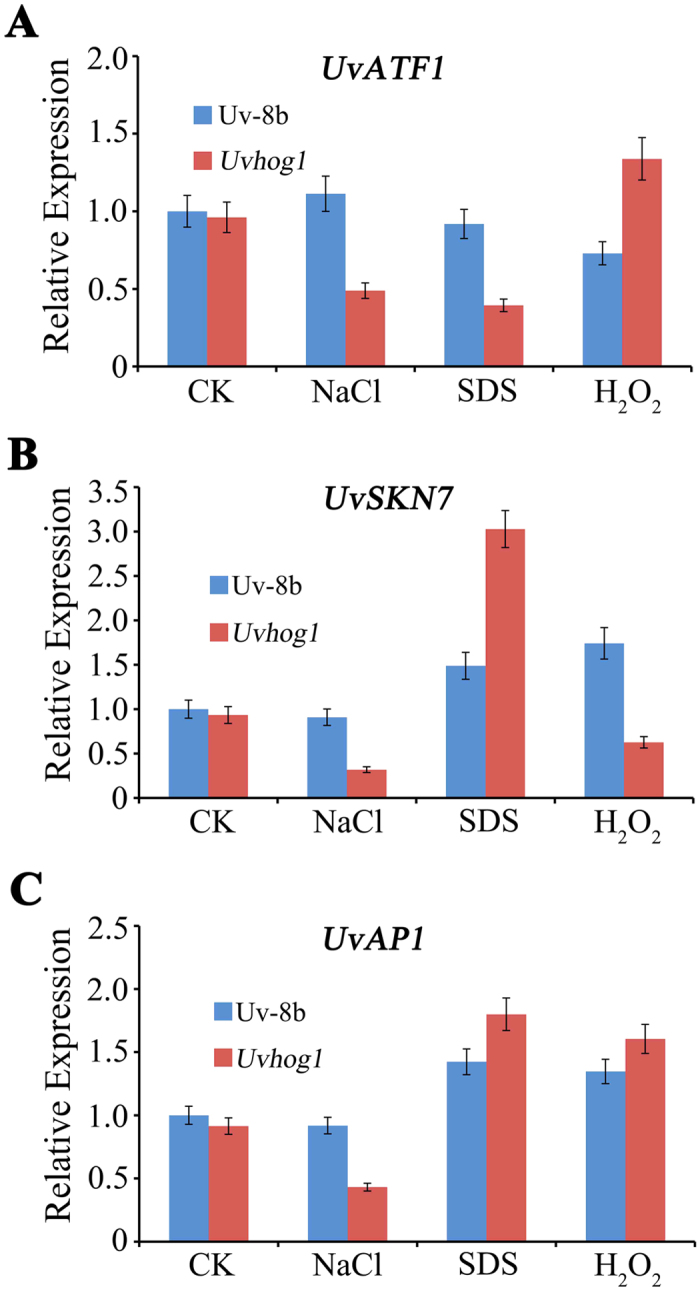
Expression profiles of *UvATF1, UvAP1,* and *UvSKN7*. RNA samples were isolated from vegetative hyphae of UV-8b and the *Uvhog1* mutant cultured in regular YT (CK) or YT with 0.5 M NaCl, 0.03% SDS, or 0.07% H_2_O_2_. The expression level of (**A**) *UvATF1*, (**B**) *UvSKN7,* and **(C)** *UvAP1* in the wild type strain cultured in regular YT was arbitrarily set to 1. Mean and standard deviations were calculated with results from three independent replicates.

The *ATF1*, *SKN7*, and *AP1* genes encode three transcription factors known to be involved in the regulation of stress-related genes in *F. graminearum* and other fungi^30^,^31^,^32^. To determine whether deletion of *UvHOG1* affects the expression of their orthologs in *U. virens*, RNA samples were isolated from hyphae of the wild-type strain UV-8b and *Uvhog1* mutant that were harvested from 2-day-old regular YT cultures and cultures treated with 0.5 M NaCl, 0.03% SDS, or 0.07% H_2_O_2_. In the wild type, the expression of *UvAP1*, *UvATF1*, and *UvSKN7* had no significant change when treated with NaCl. However, SDS treatment resulted in a 50% increase in the expression of *UvAP1* and *UvSKN7*, and H_2_O_2_ treatment caused an up-regulation of *UvSKN7* expression (Fig. 3). In the *Uvhog1* mutant, the expressions of all these three genes were reduced over two-folds by treatment with 0.5 M NaCl. Nevertheless, the expression of *UvAP1* and *UvSKN7* was up-regulated over two-folds in the presence of SDS (Fig. 3).

In comparison with the wild type, the expression of *UvATF1*, *UvSKN7*, and *UvAP1* was only slightly reduced in the *UvHog1* mutant (controls in Fig. 3). However, compared to the wild type, the expression of *UvATF1* was reduced over 50% in the presence of NaCl and SDS but up-regulated approximately two-folds when treated with H_2_O_2_ in the *Uvhog1* mutant (Fig. 3A). For *UvSKN7*, its expression in the mutant were significantly reduced when treated with NaCl but increased over two-folds in response to SDS (Fig. 3B). However, the wild type and *Uvhog1* mutant had no obvious differences in the expression level of *UvAP1* when treated with SDS or H_2_O_2_ although the presence of 0.5 M NaCl down-regulated its expression more than 50% in the *Uvhog1* mutant (Fig. 3C). These results indicate that deletion of *UvHOG1* has different effects on these transcription factor genes in *U. virens*.

### The accumulation of sorbitol and glycerol is not induced by NaCl treatment in the *Uvhog1* mutant

**Figure 4:**
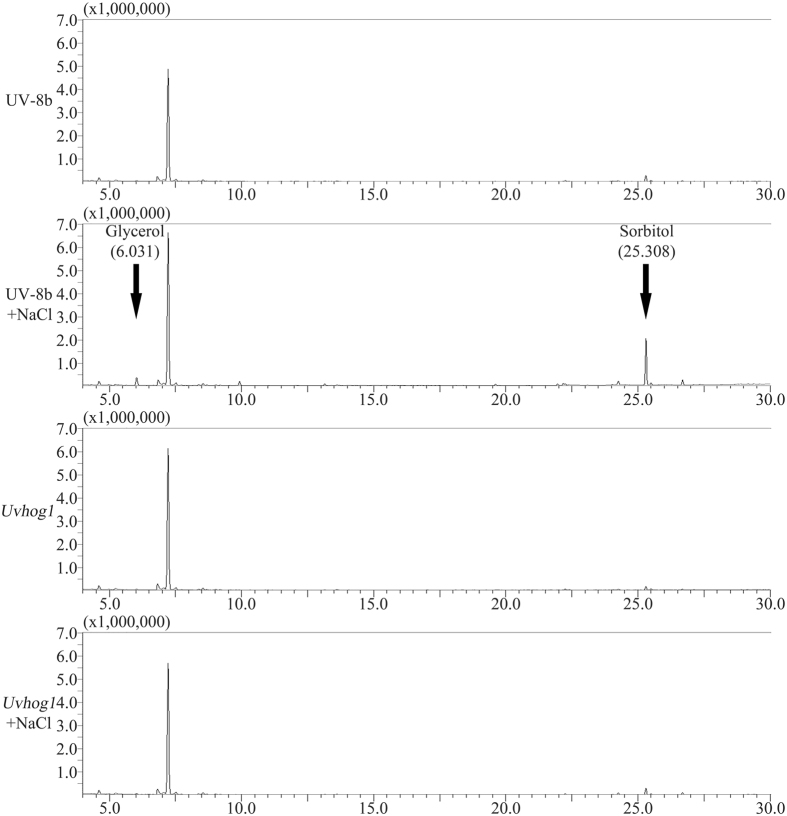
Metabolic profiles of the wild type and *Uvhog1* mutant. Vegetative hyphae were harvested from two-day-old YT cultures of the wild type strain UV-8b and *Uvhog1* mutant treated with or without 1.0 M NaCl for 1 h. Metabolites were extracted and analyzed by GC-MS. The X-axis represents the retention time (RT) in minutes. The Y-axis is the abundance of total ion current (TIC). The peaks with RT of 6.031 and 25.308 are glycerol and sorbitol, respectively. The peak with RT of 7.223 minutes is isocetane.

Glycerol, sorbitol, and other neutral compatible solutes are known to be accumulated in different fungi in response to hyperosmotic stress^33^. To determine the compatible solutes accumulated in *U. virens* in response to hyperosmotic stress, we assayed metabolites in cultures treated with or without 1.0 M NaCl. In the wild type, sorbitol that has the retention time (RT) of 25.308 min was significantly induced by NaCl treatment (Fig. 4). Glycerol (RT = 6.031 min) was also slightly induced by NaCl treatment in UV-8b. However, the production of these two compatible solutes was not or barely detectable in hyphae of the *Uvhog1* mutant treated with or without 1.0 M NaCl (Fig. 4). These results indicate that sorbitol is the main compatible solute in *U. virens* under hyperosmotic conditions, and its accumulation is under the control of the *UvHOG1* MAP kinase pathway. Glycerol accumulation is also controlled by *UvHOG1* but its contribution to adaptation to hyperosmotic stress may be not as significant as sorbitol.

### Deletion of the *UvHOG1* gene likely reduces the expression of *UvUSTA* gene

In *F. graminearum*, the *Fghog1* mutant was significantly reduced in DON production^20^. To assay the role of *UvHOG1* in mycotoxin biosynthesis, we assayed the expression of the *UvUSTA* gene, a member of the gene cluster related to ustiloxin synthesis^34^. In comparison with the wild type, the expression level of *UvUSTA* was reduced over 10-folds in the *Uvhog1* mutant after incubation for 14 days in YT medium (see Supplementary Fig. S2 online). These results indicate that *UvHOG1* may be involved in the regulation of *UvUSTA* expression in *U. virens*.

### Culture filtrates of the *Uvhog1* mutant is less inhibitory to rice seed germinating

**Figure 5:**
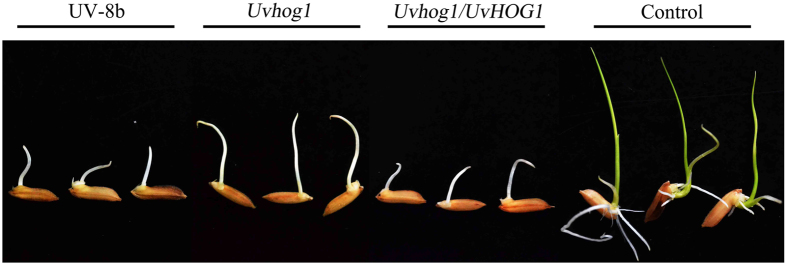
Assays for toxicity of *U. virens* culture filtrates with rice seeds. Seeds of rice cultivar YA-5A were incubated on filter papers soaked with blank control or filtrates of 5-day-old YT cultures of UV-8b, 7-day-old *Uvhog1* mutant, and 5-day-old complementary strain. Shoot and root growth were examined after incubation at 25 °C for 5 days.

To determine whether deletion of *UvHOG1* affects the production of phytotoxic compounds, we isolated the culture filtrates from YT cultures of 5-day-old wild-type strain UV-8b, 7-day-old *Uvhog1* mutant M1, and 5-day-old complementary transformant C1 as described^35^ with minor modifications. The dry weights of vegetative hyphae were quantified to show that the wild type and *Uvhog1* mutant strains had similar biomasses in these YT cultures (see Supplementary Table S3 online). When assayed with rice seeds of cultivar YA-5A, culture filtrates of the wild type, *Uvhog1* mutant, and complemented strains blocked root growth after incubation for 5 days at room temperature (Fig. 5). No visible root growth was observed in the rice seeds treated with culture filtrates of *U. virens*. Rice shoot growth also was significantly stunted in samples treated with culture filtrates of the wild type and complemented transformant. Whereas rice shoots were green and began to form the first leaf by 5 days in the water treatment control, only whitish, short shoots were observed in samples treated with culture filtrates of strains UV-8b or C1 (Fig. 5). However, rice shoot growth was less sensitive to culture filtrates of the *Uvhog1* mutant (Fig. 5). In repeated experiments, shoot growth was significantly longer in samples treated withfiltrates of 7-day-old mutant cultures than those of 5-day-old wild type or complemented transformant cultures (Table 3), suggesting that *UvHOG1* may be involved in regulating the production of phytotoxic compounds that are inhibitory to rice shoot growth during seed germination.

## Discussion

*Agrobacterium tumefaciens*-mediated transformation (ATMT) has been widely used in various fungi, including *U. virens*^36^, and it has been reported to increase the frequency of homologous recombination^9^,^37^,^38^. In this study, we failed to isolate*Uvhog1* deletion mutants with PEG-mediated protoplast transformation. Even with the ATMT approach, only one *Uvhog1* mutant was identified among the 617 hygromycin-resistant transformants screened, which was less than 0.2% for the gene replacement frequency. In separate studies in generating knockout mutants of two other genes in *U. virens*, we had similar low homologous recombination frequency with the ATMT approach. Whereas the frequency of gene replacement was 1-2% in ATMT transformants of *C. purpurea*^13^,^14^, over 50% the ATMT transformants had targeted gene deletion in *Aspergillus nidulans*^39^. Our data suggest that homologous recombination may occur at a relatively lower frequency in *U. virens*. To introduce targeted mutations or deletion more efficiently in *U. virens*, it may be helpful to generate the *UvKu70* or *UvKu80* deletion mutant, which is known to be increased in homologous recombination frequency in other fungi^40^,^41^. The other option is to use the CRISPR^42^ approach to generate targeted gene disruption mutants in *U. virens*.

The most conserved function of the Hog1 MAP kinase pathway in yeast and filamentous fungi is for regulating responses to hyperosmotic stress^17^,^43^. Therefore, it is not surprising that hyphal growth of the *Uvhog1* mutant was significantly stunted on YT plate with 0.5 M NaCl or 1 M sorbitol. In response to hyperosmotic stress, fungal hyphae often accumulate compatible solutes, including glycerol, sorbitol, and proline^33^. The accumulation of sorbitol was observed in hyphae treated with 1.0 M NaCl in the wild type but not the *Uvhog1* mutant. Although to a lesser degree, glycerol accumulation was slightly increased in response to NaCl treatment. Therefore, although glycerol may play a minor role, sorbitol is the major compatible solute accumulated by *U. virens* in response to hyperosmotic stress, which is regulated by the *UvHOG1* pathway. In *F. graminearum*, glycerol, arabitol, mannitol and sucrose are accumulated in response to hyperosmotic stress^20^. However, in *Beauveria bassiana*, erythritol and arabitol, but not glycerol and mannitol, are the major compatible solutes for adaptation to hyperosmotic stress^44^. Similarly, arabitol is a major compatible solute accumulated in *M. oryzae* under hyperosmotic stress conditions^22^. Differences in compatible solutes that are induced by NaCl treatment indicate that the *HOG1* pathway may be involve in the regulation of diverse metabolic pathways in response to hyperosmotic stress in different fungi.

In filamentous fungi, it has been shown that the Hog1 MAP kinase pathway also plays roles in regulating responses to oxidative stress^17^. In *A. fumigatus* and *B. cinerea*, vegetative growth of the mutants blocked in this conserved MAPK pathway, such as the Δ*sakA* and Δ*bcsak1* mutants, were hypersensitive to H_2_O_2_ treatment^21^,^24^. Whereas the *Fghog1* mutant was slightly increased in sensitivity to H_2_O_2_ than the wild type in *F. graminearum*^20^, the *Uvhog1* mutant had no significant changes in sensitivity to H_2_O_2_ in comparison with the wild type in *U. virens*. Therefore, the UvHog1 pathway may not play a significant role in response to oxidative stress in the rice false smut fungus. However, the *Uvhog1* mutant was also hypersensitive to cytoplasm membrane and cell wall stresses. Therefore, it is likely that the UvHog1 MAP kinase pathway also is involved in regulating responses to various environmental stresses in *U. virens*, which is similar to what has been observed in several other filamentous fungi^20^,^24^.

When cultured on regular PDA, YT, and 5× YEG media, the *Uvhog1* mutant was reduced in growth rate and produced compact colonies, suggesting a role of *UvHOG1* in hyphal growth under normal culture conditions regardless of stresses. Although this MAP kinase is dispensable for growth in fungi such as *M. oryzae* and *B. bassiana*^22^,^44^, deletion of its ortholog in *F. graminearum*, *B. cinerea, A. fumigatus*, and *Metarhizium acridum* affected hyphal growth^20^,^21^,^24^,^45^. In addition, conidiation was reduced in the *Uvhog1* mutant, which is similar to what has been observed in mutants defective in this MAP kinase pathway in *B. cinerea*, *F. graminearum* and *B. bassiana*^20^,^21^,^44^. The HOG MAP kinase pathway appears to have conserved roles in vegetative growth and conidiation in a subset of filamentous ascomycetes.

Orthologs of *ATF1, SKN7* and *AP1* transcription factors are known to play different roles in response to various stresses, fungicides resistance, and pathogenicity in different fungi^31^,^46^,^47^,^48^. For examples, the *Moatf1* mutant has increased sensitivity to H_2_O_2_^49^ but deletion of *BcATF1* has no effect on sensitivity to oxidative and hyperosmotic stresses in *B. cinerea*^50^. In *Cryptococcus neoformans*, the Δ*skn7* mutant is normal in sensitivity to H_2_O_2_ but hypersensitive to NaCl^47^. Nevertheless, the Δ*Mrskn7* mutant is not sensitive to both of oxidative and hyperosmotic stresses in *Metarhizium robertsii*^48^. In *F. graminearum*, the *Fgatf1, Fgap1,* and *Fgskn7* mutants all have increased sensitivity to oxidative stress but only *FgATF1* and *FgSKN7* are important for responses to hyperosmotic stress^30^. Although the exact functions of *UvATF1, UvSKN7,* and *UvAP1* are not clear, we found that their expression levels were all reduced over two-folds in the *Uvhog1* mutant when treated with 0.5 NaCl, indicating that *UvHOG1* may be functionally related to these transcription factors in response to hyperosmotic stress. However, deletion of *UvHOG1* had different effects on their expression in response to SDS or H_2_O_2_ treatment. Only *UvATF1* was significantly reduced in the *Uvhog1* mutant in the presence of SDS. In fact, SDS treatment increased the expression of *UvSKN7* and *UvAP1* in the mutant. Therefore, *UvHOG1* may regulate response to SDS treatment via *UvATF1*. Interestingly, under oxidative stress, *UvSKN7* expression was reduced in the *Uvhog1* mutant but increased in the wild type although the changes in its expression level were less than 2-folds. It is likely that *UvHOG1* is involved in the up-regulation of *UvSKN7* expression in response to oxidative stress in the wild type. Down regulation of *UvSKN7* in the *Uvhog1* deletion mutant may be related to possible self-regulation of *UvSKN7* in response to H_2_O_2_ treatment in *U. virens*.

Ustiloxins are mycotoxins produced by *U. virens* in false smut balls on rice plants. They can interfere cytoskeleton functions by inhibiting microtubule assembly and induce abnormal swelling of rice seedling roots^35^. The involvement of the HOG pathway in secondary metabolism has been reported in fungi such as *F. graminearum*, in which the *FgHog1* mutant was reduced in DON production^20^. In *U. virens*, the *UvUSTA* gene encodes the precursor of ustiloxins^34^ and it had reduced expressionlevel in the *Uvhog1* mutant. However, in comparison with the *U. virens* α-tubulin gene as the internal reference control, the expression level of *UvUSTA* was low in both the wide type and mutant strains, which may be related to the fact that we failed to detect the production of ustiloxins in both the wild type and mutant strains in repeated attempts. Unfortunately, conditions to stimulate ustiloxin production *in vitro* remain to be developed and optimized in *U. virens*. Although the difference in *UvUSTA* expression between the wild type and mutant was significant in repeated experiments under the experimental conditions used in this study, to determine the regulatory role of the UvHog1 MAPK pathway in ustiloxin biosynthesis, it is necessary to assay the expression level of *UvUSTA* under better culture conditions that induce ustiloxin production in the future.

The HOG MAPK pathway varies significantly among different fungal pathogens for pathogenicity. In *M. oryzae*, this pathway is dispensable for plant infection^22^. However, it is essential for virulence in several plant, insect, and human pathogenic fungi, including *M. graminicola*, *B. cinerea*, *B. bassiana*, *M acridum*, *C. neoformans*, and *Candida albicans*^19^,^21^,^44^,^45^,^51^,^52^. To determine whether *UvHOG1* is important for virulence, we attempted several times to inoculate plants of rice cultivar YA-5A with conidia isolated from the wild-type strain UV-8b, the *Uvhog1* mutant, and complemented transformant C1. Unfortunately, we failed to observe in any of the plants that were inoculated. Because infection assays with the rice false smut fungus are known to be unreliable, we assayed the toxicity of culture filtrates on rice seed germination. Interestingly, culture filtrates of both the wild type and *Uvhog1* mutant blocked root growth but only reduced rice shoot growth, suggesting that root growth is more sensitive to the inhibitory compounds present in *U. virens* culture filtrates than shoot growth. However, culture filtrates of the mutant were less inhibitory to shoot growth than those of the wild type and complemented transformant C1. It is possible that *UvHOG1* plays a role in regulating the production of inhibitory compounds or toxic secondary metabolites in *U. virens* cultures. It will be important to identify the exact chemical compounds in culture filtrates that are responsible for the inhibitory effects on rice shoot growth. One possibility is that ustiloxins are responsible for the inhibition of rice root and shoot growth by culture filtrates. However, we failed to detect ustiloxins in culture filtrates in repeated tries. Therefore, it is more likely that *U. virens* produces other secondary metabolites that are inhibitory or toxic to rice.

## Methods

### Strains and culture conditions

The wild-type strain UV-8b^2^ and all the transformants of *U. virens* generated in this study were routinely cultured on potato dextrose agar (PDA), YT (0.1% yeast extract, 0.1% tryptone, and 1% glucose)^29^, or 5xYEG (0.5% yeast extract, 0.5% pepone, and 1% glucose) plates at 25 °C. To test sensitivity against different stresses, vegetative growth was assayed after incubation at 25°C for 14 days on regular YT plates and YT with 0.5 M NaCl, 1 M sorbitol, 0.07% H_2_O_2_, 0.03% SDS (w/v), or 70 μg/ml Congo red. Conidiation was assayed with 7-day-old liquid YT cultures. Freshly harvested conidia were resuspended to 1 × 10^6^ conidia/ml in YTS (0.1% yeast extract, 0.1% tryptone, and 1% sucrose) with or without 0.3 M NaCl, or 0.03% SDS and assayed for germination after incubation for 12, 16, 18, 20 and 24 h.

### Generation of the *UvHOG1* gene replacement construct

The 1.07-kb upstream and 1.01-kb downstream flanking sequences of *UvHOG1* were amplified with primer pairs 1F/2R and 3F/4R (see Supplementary Table S1 online), respectively. The resulting PCR products were connected to the hygromycin phosphotransferase (*hph*) fragments amplified with primers HYGF/HYGR by double-joint PCR^26^. The resulting PCR product was then cloned between the *Eco*RI and *Pst*I sites on pCDW22 as pCDW23. Plasmid pCDW22 was constructed by digestion of pCAMBIA1300 (CAMBIA, Canberra, Australia) with *Xho*I and *Eco*RI, and ligated with a 0.1-kb LacZ fragment amplified with primers LacZF/LacZR (see Supplementary Table S1 online) to eliminate the *hph* resistance marker on the vector backbone. Plasmid pCDW23 was transformed into *A. tumefaciens* strain AGL-1 by electroporation.

### Transformation of *U. virens*

ATMT transformation of the wild-type strain UV-8b was performed as described^9^. For PEG-mediated transformation, conidia were harvested from 10-day-old YT cultures of the wild-type strain UV-8b by filtration through Miracloth^53^. A total of 10^8^ conidia were inoculated into 100 ml YT medium and incubated for two days at 25 °C. Hyphae were harvested by filtration with Miracloth, washed with 1.2 M KCl, and resuspended in driselase solution (15 mg/ml driselase in 1.2 M KCl). After digestion at 30 °C for 3 h with gentle shaking (60 rpm), hyphal fragments were removed by filtration and protoplasts were collected by centrifugation for 10 min at 5000 rpm. After washing twice with STC solution (20% sucrose, 50 mM Tris-HCl, 50 mM CaCl_2_), protoplasts were resuspended in STC to the final concentration of 10^8^ per milliliter. Plasmid DNA (5 μg) was mixed with 200 μl protoplasts and incubated for 25 min before mixing with 1 ml PTC solution (40% PEG8000 in STC). After incubation for 25 min, the transformation mixture was mixed with 8 ml TB3 liquid medium (3 g yeast extract, 3 g casamino acids, 20% sucrose, 1 liter H_2_O) and shaken at 60 rpm overnight at 25 °C. After mixing with 50 ml TB3 agar, an aliquot of 12 ml transformation cultures was plated out and overlaid with 15 ml of TB3 with 180 μg/ml of hygromycin B (Calbiochem, La Jolla, CA) for transformant selection.

### Complementation of the *Uvhog1* mutant

The entire *UvHOG1* gene containing the coding region and its 1.5-kb promoter and 0.5-kb terminator sequence was amplified with primers CUvHOG1/F and CUvHOG1/R (see Supplementary Table S1 online), digested with *Eco*RI and *Pst*I, and cloned into the vector pCBDW-GEN. The resulting construct, pCBDW-GEN-UvHOG1, was transformed into *A. tumefaciens* strain AGL-1 by electroporation. ATMT transformation of the *Uvhog1* mutant M1 was performed as described^9^. The resulting *Uvhog1/UvHOG1* transformants were confirmed by PCR.

### qRT-PCR assays

Vegetative hyphae harvested from two-day-old YT cultures (started with 1 × 10^6^ conidia/ ml) were further incubated in regular YT or YT with 0.5 M NaCl, 0.07% H_2_O_2_, or 0.03% SDS (w/v) for 30 min. RNA was isolated with the TRIzol reagent (Invitrogen, Carlsbad, CA). First-strand cDNA was synthesized with the Fermentas 1^st^ cDNA synthesis kit (Hanover, MD). Primers used for qRT-PCR assays were listed in Supplementary Table S1. Relative expression levels of *UvAP1, UvATF1,* and *UvSKN7* were calculated by the 2^−ΔΔCt^ method^54^ with the *U. virens* α-tubulin gene as the endogenous reference^55^. Data from three biological replicates were used to calculate the mean and standard deviation.

### Assays for compatible solutes

Freshly harvested conidia were resuspended to 1 × 10^6^ conidia/ml in YT with sucrose replacing glucose and incubated for 2 days at 25 °C. Vegetative hyphae were then harvested and divided into two halves. One half was incubated in regular YT and the other half was incubated in YT with 1.0 M NaCl at 25 °C for 1 h. Hyphae were then harvested, rinsed with distilled water, ground in liquid nitrogen, and dried for 24 h in a freeze dryer. Six milligrams of ground hyphae were transferred into a 4 ml auto sampler vial, suspended in 2 ml methanol, and incubated overnight at 25 °C. After centrifugation at 4,000 rpm for 10 min, 100 μl of the supernatant was transferred to a conical vial and dried under a gentle nitrogen stream. The content was then re-suspended in 100 μl of 1 M HCl, incubated at 50 °C for 1 h, dried under a nitrogen stream as described^56^,^57^. The solid extract was re-suspended in 100 μl of TMSI:TMCS (100:1), incubated at 37 °C for 1 h, then mixed with 300 μl of isooctane and 300 μl water. After the aqueous and organic layers were completely separated, the top organic phase was analyzed with a GCMS-QP2010 (Shimadzu, Japan) in the scan mode (scanning from m/z 40 to 650). Helium was used as the carrier gas at a constant flow rate of 1 mL/min through an Rxi-5 ms (30 m × 60.25 mm, 0.25 μm) capillary column (Restrek, Bellefonte, USA) with the injector temperature of 260 °C and split ratio of 1:5.

### Toxicity assays with culture filtrates

Hyphae were collected by filtration through two layers of Miracloth (Calbiochem, La Jolla CA, USA) from 5-day-old YT cultures of the wild type and complemented transformant C1 and 7-day-old liquid YT culture of the *Uvhog1* mutant and measured for dry weights after dehydration in a freezer dryer for 18 h. Culture filtrates were then centrifuged at 7, 000 rpm for 5 min to collect the supernatants. Seeds of rice cultivar YA-5A were incubated on filter papers soaked with the resulting culture filtrates at 25 °C under 14 h light/10 h dark. Shoot and root growth were measured after incubation for 5 days.

## Additional Information

**How to cite this article**: Zheng, D. *et al.* *UvHOG1* is important for hyphal growth and stress responses in the rice false smut fungus *Ustilaginoidea virens. Sci. Rep.* **6**, 24824; doi: 10.1038/srep24824 (2016).

## Supplementary Material

Supplementary Information

## Figures and Tables

**Table 1:** Phenotypes of the *Uvhog1* mutant in vegetative growth and conidiation.

Strains	Colony Diameter (mm)*			Conidiation (×10^5^/ml)**
	PDA	YT	5xYEG	
UV-8b	43.0 ± 0.1^A^	48.0 ± 0.2^A^	41.0 ± 0.1^A^	8.2 ± 0.4^A^
M1	36.0 ± 0.3^B^	38.0 ± 0.4^B^	35.0 ± 0.2^B^	1.8 ± 0.1^B^
C1	38.2 ± 0.4^B^	48.0 ± 0.1^A^	43.0 ± 0.1^A^	8.5 ± 0.7^A^

^*^Colony diameter was measured with petri plate cultures (Φ9 CM) after incubation for 14 days.

^**^Conidiation was assayed with 7-day-old YT cultures.

Mean and standard deviation were calculated from three independent replicates. Different letters mark significant differences by ANOVA analysis (P = 0.05).

**Table 2:** Reduction in hyphal growth of the *Uvhog1* mutant by different stresses.

Strains	Percentage of reduction in colony diamether (%)*				
	0.5 M NaCl	1 M Sorbitol	0.07% H_2_O_2_	0.03% SDS	70 μg/ml Congo red
UV-8b (wild type)	72.5 ± 0.3^A^	76.0 ± 0.9^A^	71.9 ± 0.4^A^	33.3 ± 0.2^A^	16.7 ± 1.1^A^
M1 (*Uvhog1*)	100** ^B^	99.7 ± 0.0^B^	73.7 ± 0.2^B^	100** ^B^	44.7 ± 0.4^B^
C1 (*Uvhog1*/*UvHOG1*)	71.6 ± 0.6^A^	71.9 ± 0.4^A^	69.8 ± 0.2^A^	37.5 ± 0.2^A^	20.8 ± 0.2^A^

^*^Colony diameter was measured after incubation for 14 days on regular YT medium or YT with different chemicals. For each strain, the percentage of reduction in growth by different stresses was estimated in comparison with its growth on regular YT medium after deducting the diameter of the original inoculum (Φ 8 mm). Mean and standard deviation were calculated from three replicates. Different letters mark significant differences by ANOVA analysis (P = 0.05).

^**^no visible growth.

**Table 3:** Inhibitory effects of culture filtrates of *U. virens* on rice seed germination.

Strains	Shoot growth (mm/5 day)*	Root growth (mm/5 day)*
Water	23.4 ± 6.4^A^	10.9 ± 8.6
UV-8b^a^	11.5 ± 5.8^B^	N/D**
M1^b^	19.4 ± 4.5^C^	N/D
C1^a^	12.6 ± 5.4^B^	N/D

^*^Root and shoot growth were measured after incubation of rice seeds on filter papers with culture filtrates for 5 days. Mean and standard deviation were calculated from three independent replicates with 50 seeds for each treatment. Different letters mark significant differences by ANOVA analysis (P = 0.05).

^**^N/D, not detectable.

^a^Culture filtrates harvested from 5-day-old YT cultures.

^b^Culture filtrates harvested from 7-day-old YT cultures.
