# Supplementary material for: UvHOG1 is important for hyphal growth and stress responses in the rice false smut fungus Ustilaginoidea virens
Source: Sci Rep. 2016 Apr 20;6:24824. doi: 10.1038/srep24824 (PMC4837404; doi:10.1038/srep24824)
Supplement: Supplementary Information [file srep24824-s1.pdf]

UvHOG1 is important for hyphal growth and stress responses in the rice false smut fungus *Ustilaginoidea virens*

Dawei Zheng[1], Yi Wang[1], Yu Han[1], Jin-Rong Xu[2], and Chenfang Wang[1*]

[1] State Key Laboratory of Crop Stress Biology for Arid Areas, College of Plant Protection, Northwest A&F University, Yangling, Shaanxi 712100, China.

[2] Dept. of Botany and Plant Pathology, Purdue University, West Lafayette, IN 47907. USA.

***Corresponding author:** Chenfang Wang
Tel: 86-029-8708-1270
Email: wangchenfang@nwsuaf.edu.cn

Supplementary Figure S1

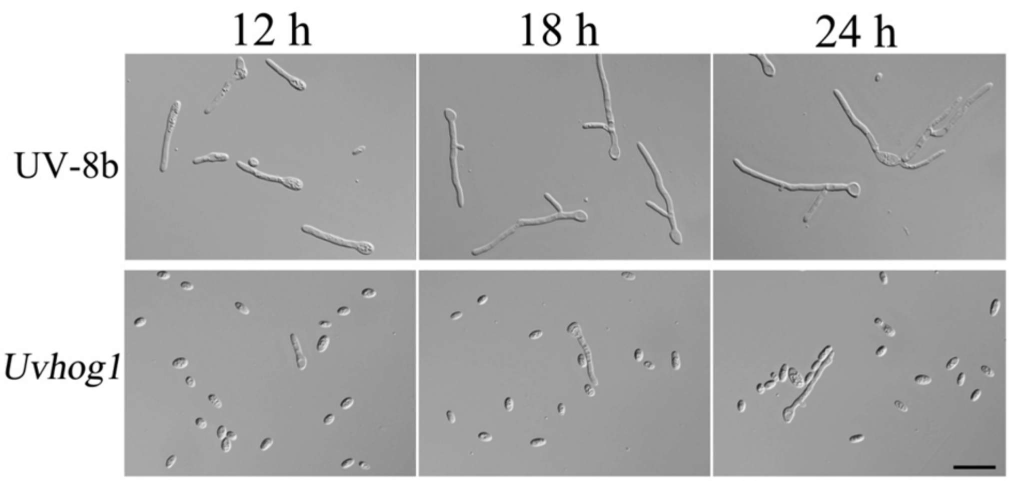

Figure S1. Conidia of the wild type strain UV-8b and the *Uvhog1* mutant were cultured in YTS with 0.03% SDS for 12, 18, and 24 h. Bar = 20 μm.

Supplementary Figure S2

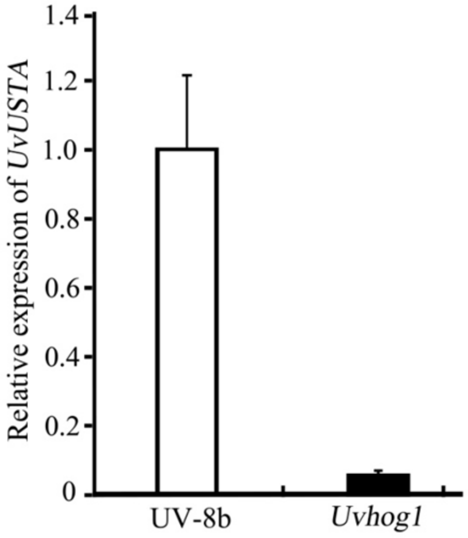

Figure S2. Assays for the expression of *UvUSTA* by qRT-PCR. RNA samples were isolated from vegetative hyphae of the wild type UV-8b and *Uvhog1* mutant after incubation for 14 days in liquid YT cultures at 25°C. The expression level of *UvUSTA* in 14-day-old wild-type culture was arbitrarily set to 1. Mean and standard deviations were calculated with results from three independent replicates.

Supplementary Table S1. Primers used in this study

Primers	Sequences (5` to 3`)
UVHOG1/F5	GATAGTACCGAGTAGACACGTAGCC
UVHOG1/R6	GCACCTCAAGCACGAGAATGTAG
UVHOG1/R2	TTGACCTCCACTAGCTCCAGCCAAGCCACGATCAACAAGCCGACCATG
UVHOG1/F1	GTTCTCGACAAGCAGGGAATCG
UVHOG1/F3	GAATAGAGTAGATGCCGACCGCGGGTTGTAACGGTTTTGCGAGTGGTT
UVHOG1/R4	CTGGGATTTGACTGCCTTTGG
UVHOG1/NF-ERI	GGAATTCCAGTGCGGTTCGACGACG
UVHOG1/NR-PSTI	AACTGCAGGAGTCCTCCAAGGGAATGAATGGT
LacZF	CCGCTCGAGCCCCAGGCTTTACACTTTATGCT
LacZR	CAAGGCGATTAAGTTGGGTAACG
HYG/F	GGCTTGGCTGGAGCTAGTGGAGGTCAA
HYG/R	AACCCGCGGTCGGCATCTACTCTATTC
H856F	GTCGATGCGACGCAATCGT
H855R	GCTGATCTGACCAGTTGC
CUvHOG1/F	GGAATTCCGCGTCGTTGTCTTTCACGA
CUvHOG1/R	AACTGCAGCTTCTCAGCATCTCAGAGGACG
qUVHOG1F1	GGTACGGAGCAAGATATTCG
qUVHOG1R2	TCATCAACACCATCGCAAG
qUV*SKN7*F1	TCCACCTCGCAGTCCATC
qUV*SKN7*R2	CGGCTCCAGTCTCCAGTA
qUV*AP1*F1	ACTACCGAGAATCGCAGGAA
qUV*AP1*R2	GGTGTTGAAGGGCGTGAAG
qUV*ATF1*F1	TCGCCCTTGGCAATGTG
qUV*ATF1*R2	ACCGTGTATGACGAAGAGAA
qUVUSTF	AGTTTTCGCTCATCTCCATCTTG
qUVUSTR	CGCTTTGCCATTACATTCTCCT
UV-a-tubulin-1/F	AGGTTGCGTTGAAGGAGGTT
UV-a-tubulin-1/R	GAGGTGGAGTTGCCGATAAA

Supplementary Table S2. Percentage of conidium germination of the wild type and *Uvhog1* mutant by different stresses (%)

Strains	0.3 M NaCl*			0.03% SDS*		
	16 h	20 h	24 h	12 h	18 h	24 h
UV-8b	72.1±1.9^{A}	84.5±2.6^{A}	95.2±1.4^{A}	76.2±1.2^{A}	94.5±1.9^{A}	98.1±1.1^{A}
M1	3.2±1.3^{B}	4.8±0.9^{B}	5.0±0.8^{B}	2.5±1.0^{B}	2.8±0.6^{B}	3.2±0.3^{B}
C1	72.4±1.8^{A}	84.7±4.4^{A}	95.4±2.5^{A}	74.6±1.7^{A}	94.4±1.8^{A}	98.4±0.7^{A}

* Conidia of UV-8b and the *Uvhog1* mutant were incubated in YTS with 0.3 M NaCl or 0.03% SDS for different times. At least 200 conidia were calculated in each independent repeat. Mean and standard deviation were calculated from three replicates. Different letters mark significant differences by ANOVA analysis (P = 0.05).

Supplementary Table S3. Dry weights of hyphae in the wild-type strain UV-8b and *Uvhog1* mutant

Strains	Dry Weight (mg) *
Uv-8b (WT)	58.0±1.0
M1 (*Uvhog1*)	58.5±2.3
C1 (*Uvhog1*/*UvHOG1*)	58.2±1.5

* Dry weights of hyphae harvested from 5-day-old YT cultures of the wild-type strain and complemented transformant and 5-day-old YT culture of the *Uvhog1* mutant. Mean and standard deviation were calculated from three independent replicates.
